# Potential profile analysis and influencing factors of physical nurse job embedding

**DOI:** 10.3389/frhs.2026.1700816

**Published:** 2026-02-23

**Authors:** Gan Jingjing, Zhang Zhi, Cao Yueping

**Affiliations:** 1 Health Management Center, Shanghai Public Health Clinical Center, Shanghai, China; 2 Department of Ultrasound Medicine, Shanghai Public Health Clinical Center, Shanghai, China

**Keywords:** influencing factor, job embedding, PCQ, physical examination nurse, potential profile analysis

## Abstract

**Objective:**

This study aims to analyze the latent profiles of job embeddedness among physical examination nurses and their influencing factors, providing a scientific basis for nursing talent management.

**Methods:**

Using convenience sampling, 150 physical examination nurses from Shanghai Public Health Clinical Center were surveyed. Data were collected using a general information questionnaire, the Job Embeddedness Scale, the Nurse Retention Intention Scale, the Nurse Work Environment Scale, and the Psychological Capital Questionnaire (PCQ). Latent profile analysis and multivariate logistic regression analysis were employed.

**Results:**

The average score of job embeddedness among nurses was 69.89 ± 11.67, revealing three latent profiles: low (26.7%), medium (31.4%), and high (41.9%). Multivariate analysis indicated that annual income, promotion satisfaction, retention intention, and psychological capital (PCQ) were the main influencing factors (*p* < 0.05).

**Conclusion:**

Job embeddedness among physical examination nurses exhibits heterogeneity. It is recommended to enhance job embeddedness and stabilize the nursing workforce through comprehensive measures, such as establishing standardized management systems, optimizing nursing management structures, and ensuring reasonable income.

## Introduction

Nursing shortages have emerged as a significant challenge to global healthcare systems (1). Nurses working in health examination centers may experience empathy fatigue after prolonged exposure to the diverse demands and emotional states of patients, manifesting as emotional depletion, persistent exhaustion, and diminished professional engagement, often culminating in thoughts of resignation. Notably, nurses in health examination departments are at risk of developing mental health issues such as anxiety and depression due to workplace pressures and interpersonal conflicts, contributing to elevated turnover rates.

To mitigate the high levels of stress and turnover observed among nurses in health examination departments, this study employs latent profile analysis to examine fundamental aspects of their working conditions, including the work environment, job-related sacrifices, and occupational stress. Concurrently, the research framework of job embeddedness is incorporated to investigate various factors associated with employee turnover. Job embeddedness, defined as the aggregate of positive factors that discourage individuals from leaving their employment, serves as a key variable for predicting and explaining turnover behavior (2). Latent profile analysis is a person-centered statistical method that identifies latent subgroup structures within populations, allowing for the classification of distinct subgroups based on differing characteristics across variables (3).

In their professional roles, nurses in health examination departments should actively cultivate positive relationships and collegial friendships with colleagues, supervisors, and team members. Regular participation in hospital- and department-sponsored social activities is also encouraged. Furthermore, it is essential for nurses to assess their alignment with their specific job roles, departmental expectations, and the broader nursing culture of the institution. Professionally, this entails proactive efforts to enhance specialized skills, pursue further education, establish meaningful career objectives, and diligently fulfill job responsibilities. Beyond work duties, engagement in hospital-based cultural and social initiatives should be prioritized to foster familiarity with the organizational culture.

In terms of social compatibility, nurses should strive to balance professional obligations with family life, seeking harmony between career fulfillment and personal well-being. Research has identified several factors that influence job embeddedness among nurses, including the foundation of high-quality nursing care, nurse retention intention, the competencies and leadership styles of nursing supervisors, annual income, institutional accreditation levels, and satisfaction with career advancement opportunities (4). Therefore, this study aims to conduct a latent profile analysis of job embeddedness among nurses in health examination settings, while examining the characteristics and associated factors related to turnover. The findings are intended to provide evidence to guide managerial strategies for supporting and developing nursing talent in health examination departments.

## Materials and methods

### Research subjects

A convenience sampling method was employed to select 150 nurses from the Physical Examination Department of Shanghai Public Health Clinical Center as study participants between June 2023 and June 2024. According to Kendall's principle, the sample size for analysis should be 5–10 times the number of independent variables. With 24 independent variables in this study and accounting for a 10% rate of invalid questionnaires, the calculated required sample size ranged from 134 to 267 participants. The actual inclusion of 150 nurses met the sample size requirement.

Inclusion criteria were as follows: possession of a valid nurse practicing certificate; current employment in physical examination nursing; work experience exceeding 6 months; voluntary participation in the survey.

Exclusion criteria included nurses on sick leave, personal leave, maternity leave, or those in rotational training positions.

### Research methods

General Information Questionnaire(GIQ) This study was based on literature research and designed by the relevant nursing staff after discussion. It includes age, position, education, fertility circumstance, working years, annual income, family economic burden, health status, personal promotion satisfaction, and work environment evaluation.

Global Job Embeddedness Items (GJEI) was compiled by Mitchell et al. (2), and translated into Chinese by Qian Ying (5). It included four dimensions: organizational matching, organizational emotion, community harmony and professional sacrifice. The Likert 5-point scale was used, ranging from “strongly disagree” and “strongly agree” from 1 to 5 points. The total score ranged from 20 to 100, with higher scores indicating higher levels of job embeddedness. In this study, Cronbach's *α* coefficient of the scale was 0.937.

Exploratory latent profile analysis was conducted using Mplus 8.7 software to categorize health examination nurses based on their job embeddedness scores. The analysis began with a one-class model and incrementally increased the number of classes until the model fit indices reached an optimal state. The model fit indices included:

Information evaluation criteria:

Log-likelihood ratio test (LRT)
Akaike Information Criterion (AIC)
Bayesian Information Criterion (BIC)
Adjusted BIC (aBIC)
Lower values of AIC, BIC, and aBIC indicate better model fit.

Classification evaluation criteria:

Entropy, where values closer to 1 indicate higher classification accuracy.

Likelihood ratio tests:

Lo-Mendell-Rubin adjusted likelihood ratio test (LMR)
Bootstrapped likelihood ratio test (BLRT)

These tests compare the fit between models with k-1 and k classes. *P* < 0.05 indicates that the k-class model significantly outperforms the k-1-class model.

### Nurses' intention to remain employed scale

Nurses' Intention to Remain Employed Scale (6) is a single dimension with 6 items, Likert 5-point scale is used to score, 1 = never possible, 5 = very possible, the total score is 6–30 points, Higher scores indicate greater intention to stay. The Cronbach's *α* coefficient of the scale was 0.797.For the nurse work Environment Scale, the Chinese version of the Practice Environment Scale (PES) translated by Wang Li et al. (7) was adopted. There were 5 dimensions including nurses’ participation in hospital affairs (8 items), high-quality nursing service foundation (9 items), ability and leadership style of nursing managers (4 items), sufficient manpower and material resources (4 items) and medical cooperation (3 items), with a total of 28 items. A 4-point scale was used, with 1 = completely disagree and 4 = completely agree. The total score ranged from 28 to 112, with higher scores indicating better nursing work environment. The Cronbach's *α* coefficient of the scale was 0.875.

Psychological Capital Questionnaire (PCQ) was compiled by Luthans et al. (8) and translated by He Zhonghua et al. (9,10). Four dimensions including self-efficacy (6 items), hope (6 items), resilience (5 items) and optimism (3 items) were included. Likert 6-point scale was used, ranging from 1 to 6 points from “strongly disagree” to “strongly agree”. The total score ranged from 20 to 120, with higher scores indicating higher levels of nurses' psychological capital. The Cronbach's *α* coefficient of the scale in this study was 0.963.

### Data collection methods

Questionnaire Star was used to conduct the questionnaire survey. The researchers contacted the person in charge of the physical examination department of Shanghai Public Health Clinical Center and distributed the questionnaire, fully explaining the significance, purpose, content and precautions of the survey, and invited the person in charge of the physical examination center to send the link of the questionnaire to the nurses in the department to fill in. A total of 160 questionnaires were collected, and 150 valid questionnaires were collected, with an effective recovery rate of 93.75%.

### Statistical methods

Statistical analyses were performed using SPSS 26.0 software. Continuous variables following a normal distribution were expressed as mean ± standard deviation, while categorical data were presented as frequencies, percentages, or rates. Exploratory latent profile analysis was conducted with Mplus 8.7 software to categorize nurses' job embeddedness based on varying scores. Multivariate logistic regression analysis was employed to identify factors influencing job embeddedness among the nurses. All measurement tools were adapted and culturally validated for the specific research context. Statistical significance was set at *p* < 0.05.

## Results

### Results of latent profile analysis of job embeddedness of physical examination nurses

The results of this study showed that the score of job embeddedness of 150 physical examination nurses was (69.89 ± 11.67). One to five latent category models were constructed based on the four dimensions of the job embeddedness Scale for Physical examination nurses (Table 1). It can be seen from the table that the AIC, BIL and aBIC indexes gradually decreased with the increase of the number of models. When the number of models was 3, the entropy value was closest to 1, which was 0.975, and both LMR and BLRT tests were statistically significant (*p* < 0.001). After comprehensive comparison, model 3 with three categories was selected as the best fitting model.

**Table 1:** Fitting results of latent profile analysis model of job embeddedness for physical examination nurses (*n* = 150).

Model of categories	AIC	BIC	aBIC	Entropy	LMR (*P*)	BLRT (*P*)	Probability of class
1	11,258.135	11,456.324	11,126.421				
2	10,158.050	10,235.145	10,169.321	0.959	<0.001	<0.001	0.369/0.631
3	9,617.431	9,786.214	9,756.658	0.975	<0.001	<0.001	0.267/0.314/0.419
4	9,531.395	9,635.123	9,658.345	0.967	0.205	<0.001	0.079/0.270/0.083/0.568
5	9,443.431	9,501.645	9,547.258	0.961	0.014	<0.001	0.083/0.245/0.104/0.137/0.432

**Figure 1:**
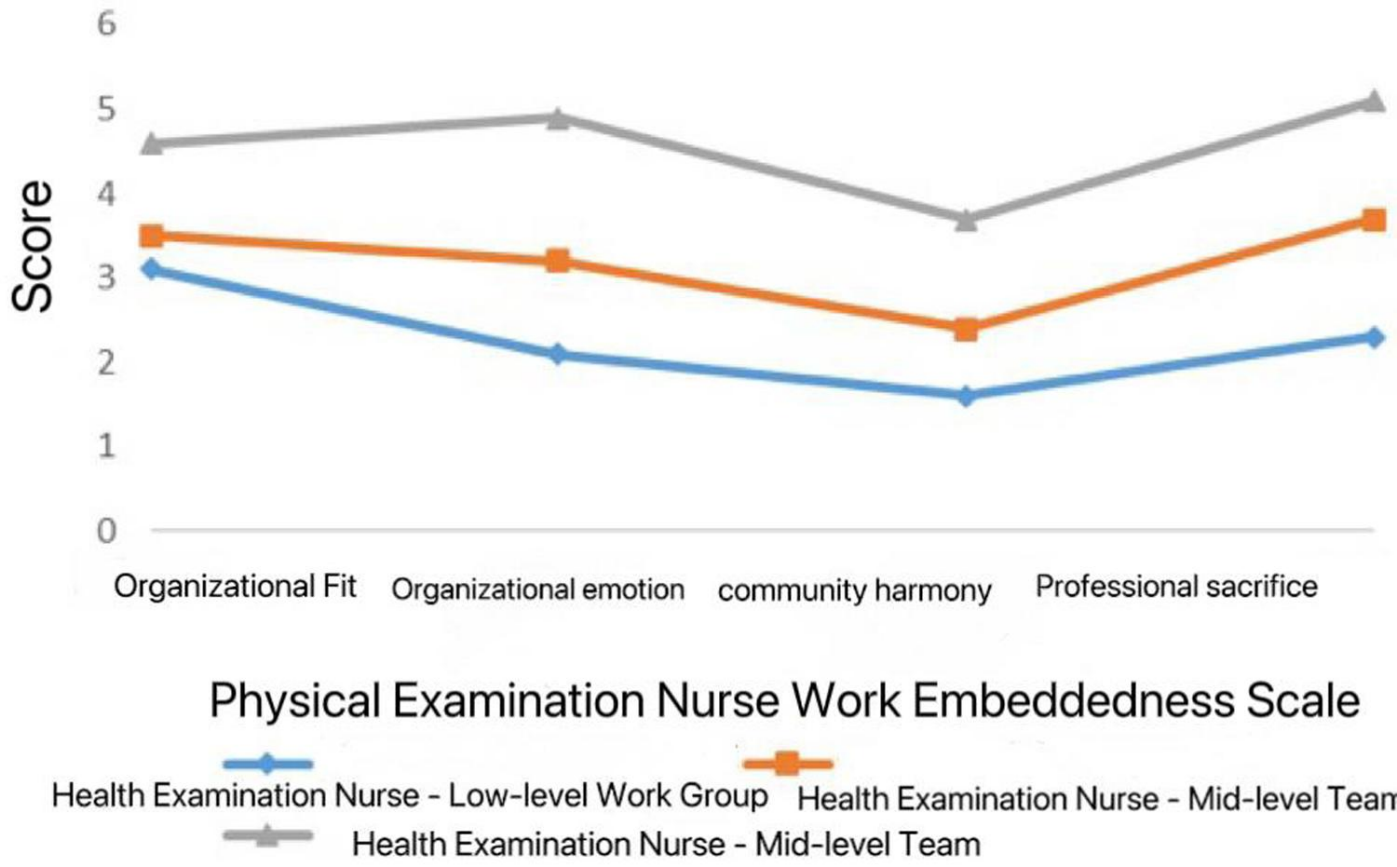
Potential profile of job embeddedness for physical examination nurses.

Based on model 3, the mean scores of each category in the job embeddedness scale for physical examination nurses, and are named according to the explicit characteristics of the scale item scores (Figure 1). There were 40 cases (26.7%) in the low, 47 cases (31.4%) in the medium and 63 cases (41.9%) in the high embeddedness of physical examination nurses.

### Results of single factor analysis of the potential profile of job embeddedness of physical examination nurses

According to the single factor results, there were statistically significant differences in age, fertility, annual income, personal promotion satisfaction, nurses' intention to stay and PCQ scores among different potential profiles of job embeddedness of physical examination nurses (*p* < 0.05) (Table 2).

**Table 2:** Univariate analysis of potential categories of job embeddedness in general data of physical examination nurses (*n* = 150).

Items	total (*n* = 150)	low-level job embedding group (*n* = 40)	medium-level job embedding group (*n* = 47)	high-level job embedding group (*n* = 63)	Testing Statistic	*P*
Age					3.530	0.009
<25	4	2 (5.00)	1 (2.13)	1 (1.59)		
25∼35	10	8 (20.00)	1 (2.13)	1 (1.59)		
36∼40	76	17 (42.50)	30 (63.83)	29 (46.03)		
40∼49	40	7 (17.50)	14 (29.79)	19 (30.16)		
50∼	20	6 (15.00)	1 (2.13)	13 (20.63)		
Post					1.664	0.193
Junior professional title	66	23 (57.50)	21 (44.68)	22 (34.92)		
Senior professional title	49	11 (27.50)	15 (31.91)	23 (36.51)		
General nurse	35	6 (15.00)	11 (23.40)	18 (28.57)		
Education background					−0.004	0.997
Bachelor degree or above	124	33 (82.50)	39 (82.98)	52 (82.54)		
College or below	26	7 (17.50)	8 (17.02)	11 (17.46)		
Fertility status					3.450	0.001
Fertility	139	32 (80.00)	45 (95.74)	62 (92.54)		
Not giving birth	11	8 (20.00)	2 (4.30)	1 (1.49)		
Length of service					0.883	0.451
1∼5	9	4 (7.50)	2 (4.26)	3 (22.22)		
5∼10	25	8 (22.2)	9 (10.64)	8 (17.46)		
10∼15	73	20 (37.50)	21 (44.68)	32 (23.81)		
15∼20	43	8 (27.50)	15 (36.17)	20 (30.16)		
Annual income (ten thousand yuan)					−1.217	0.026
5∼10	89	26 (65.00)	31 (65.96)	32 (50.79)		
10∼15	61	14 (35.00)	16 (34.04)	31 (49.21)		
Whether there is a family financial burden					−1.285	0.201
yes	78	26 (65.00)	31 (65.96)	21 (50.79)		
no	72	14 (35.00)	16 (34.04)	42 (49.21)		
Whether health status has an impact on work					−0.723	0.471
yes	87	26 (65.00)	31 (65.96)	30 (50.79)		
no	63	14 (35.00)	16 (34.04)	33 (49.21)		
Whether health status has an impact on work					2.726	0.007
Satisfied with	95	13 (32.50)	31 (65.96)	51 (80.95)		
Not satisfied with	55	27 (67.50)	16 (34.04)	12 (19.05)		
Work environment assessment					−2.412	0.231
good	84	30 (65.00)	31 (65.96)	23 (50.79)		
In general	66	27 (35.00)	24 (34.04)	15 (49.21)		
Nurses’ intention to stay was scored[($\bar{x}$±s)]	22.51 ± 4.35	19.25 ± 3.21	20.10 ± 2.98	21.35 ± 3.18	6.330	0.002
PES score[($\bar{x}$±s)]	83.91 ± 13.51	79.76 ± 11.89	80.19 ± 12.97	82.78 ± 13.42	1.001	0.571
PCQ score[($\bar{x}$±s)]	86.94 ± 15.87	79.89 ± 14.29	80.96 ± 14.15	81.39 ± 15.67	6.794	0.001
Self efficacy	25.69 ± 6.34	19.68 ± 3.14	21.37 ± 5.46	22.69 ± 6.01	5.014	0.013
Hope	24.39 ± 5.69	17.69 ± 3.01	20.14 ± 2.34	23.47 ± 4.37	4.985	0.016
Toughness	19.67 ± 3.69	14.67 ± 1.67	15.98 ± 2.03	18.47 ± 1.38	5.137	0.009
Optimism	17.19 ± 2.67	11.34 ± 1.06	14.64 ± 2.01	16.37 ± 1.54	6.387	0.004

### Results of multivariate analysis of job embeddedness potential profile of physical examination

Nurses Taking age, fertility, annual income, personal promotion satisfaction, nurses' intention to stay score and PCQ score with statistical significance in univariate analysis as independent variables and taking three potential categories of job embeddedness of physical examination nurses as dependent variables, multivariate Logistic regression analysis was conducted (Table 3).Multivariate Logistic analysis showed that compared with C2 group, physical examination nurses with lower annual income, intention to stay and PCQ score were more likely to enter C1 group, and physical examination nurses with higher satisfaction with personal promotion were more likely to enter C2 group. Compared with group C3, physical examination nurses with lower annual income, lower scores of intention to stay and PCQ were more likely to enter group C1, and higher personal promotion satisfaction were more likely to enter group C3. Compared with group C3, physical examination nurses with moderate annual income, low intention to stay and relatively low PCQ score may enter group C2, and physical examination nurses with higher satisfaction with personal promotion are more likely to enter group C3 (Table 4).

**Table 3:** Assignment of variables.

Serial number	Variable label	Assignment description
1	Age	<25 = 1; 25∼29 = 2; 30∼39 = 3; 40∼=4
2	Fertility status	Fertility =1; No birth =2
3	Personal promotion satisfaction	Satisfaction =1; Dissatisfaction =2
4	Annual income	<5 = 1; 5∼=2; 10∼=3; >20 = 4
5	Nurses’ intention to stay was scored	original value
6	PCQ Score	original value
7	Physical examination nurses work embedded in latent categories	Low job embeddedness group =1; Medium job embeddedness group =2; Higher job embeddedness group =3

**Table 4:** Multivariate logistic regression analysis of job embeddedness of physical examination nurses in three latent profiles.

Items	*β*	*SE*	Wald*χ^2^*	*P*	*OR*	95%*CI*
C1 vs. C2a						
Term of constant	8.612	0.813	71.236	<0.001	—	—
Age	0.045	0.006	5.798	0.053	1.990	1.493∼2.987
Fertility status	−1.118	0.323	5.519	0.059	0.084	0.011∼0.964
Personal promotion satisfaction	−1.321	0.468	5.698	0.001	0.650	0.134∼0.946
Annual income	0.031	0.006	5.798	0.013	2.980	1.964∼4.996
Nurses’ intention to stay was scored	0.371	0.183	5.698	0.001	2.950	1.934∼3.996
PCQ Score	3.118	0.523	13.965	0.001	4.058	4.032∼4.762
Self efficacy	1.697	0.019	9.647	0.014	2.039	1.058∼3.124
Hope	1.547	0.014	8.746	0.015	1.097	1.011∼1.187
Toughness	2.103	0.167	9.674	0.011	2.014	2.001∼2.947
Optimism	2.147	0.178	10.357	0.004	2.369	2.014∼3.012
C1 vsC3a						
Term of constant	−16.342	1.561	136.214	<0.001	—	—
Age	−0.057	0.017	3.240	0.124	0.059	0.015∼0.144
Fertility status	3.156	0.570	4.339	0.131	3.994	1.976∼6.014
Personal promotion satisfaction	−2.529	0.281	3.532	0.002	0.697	0.012∼0.837
Annual income	1.129	0.398	4.695	0.024	2.324	1.136∼3.730
Nurses’ intention to stay was scored	2.371	0.232	5.122	0.002	4.370	2.037∼9.369
PCQ Score	3.053	1.486	4.695	0.001	5.055	4.124∼9.479
Self efficacy	1.687	0.015	8.369	0.016	1.154	1.063∼2.164
Hope	1.367	0.021	7.348	0.014	1.031	1.001∼1.369
Toughness	1.964	0.154	8.564	0.011	1.574	1.001∼2.947
Optimism	2.697	0.186	10.214	0.006	2.647	2.014∼3.012
C2vsC3^b^						
Term of constant	11.610	1.236	86.325	<0.001	—	—
Age	0.051	0.035	4.468	0.117	1.950	1.934∼2.996
Fertility status	3.118	0.523	6.965	0.311	3.431	2.632∼5.762
Personal promotion satisfaction	−1.301	0.158	3.532	0.001	0.156	0.032∼0.733
Annual income	4.163	0.978	5.467	0.007	6.855	6.278∼11.363
Nurses’ intention to stay was scored	0.671	0.231	5.345	0.002	1.690	1.483∼2.987
PCQ Score	2.481	2.013	4.510	0.009	3.784	1.011∼5.964
Self efficacy	1.013	0.007	6.369	0.024	1.034	1.002∼1.647
Hope	1.031	0.009	7.549	0.026	1.065	1.001∼1.369
Toughness	1.374	0.034	8.147	0.031	1.457	1.016∼1.978
Optimism	1.579	0.101	9.014	0.010	2.014	2.001∼2.984

C1 is the low-level job embedding group of physical examination nurses, C2 is the medium-level job embedding group of physical examination nurses, and C3 is the high-level job embedding group of physical examination nurses. a takes C1 as the reference object; b takes C2 as the reference object; - indicates that the constant term has no such value.

Annual income directly reflects nurses’ professional value and livelihood security. Inadequate income may lead nurses to question organizational fairness and their own sense of value, thereby weakening their financial connection to their work.Satisfaction with promotion not only reflects nurses' recognition of career development prospects but also demonstrates the organization's acknowledgment of their professional capabilities and the fairness of its systems. High promotion satisfaction indicates that nurses perceive a strong alignment between their personal growth and the organization's development, making them more willing to invest emotional and professional efforts into long-term career advancement.

Retention intention represents a combination of nurses' emotional commitment to the organization and the perceived barriers to leaving. Low retention intention often signals a state of “psychological detachment,” which may be associated with work-related stress, burnout, or a lack of belonging.Psychological capital encompasses psychological resources such as self-efficacy, resilience, hope, and optimism. It serves as an internal driving force for nurses to cope with stress and maintain professional resilience.The influence of age and parenthood status on job embeddedness is mediated or moderated by other variables, such as income, promotion satisfaction, and psychological capital.

## Discussion

### The job embeddedness of physical examination nurses is at an above-average level

This study revealed that the job embeddedness level of physical examination nurses scored (69.89 ± 11.67) points, indicating an overall moderately high level. This score is lower than that reported by Liang Baofeng et al. (11) for clinical nurses and by Hou Dandan (12) for specialized operating room nurses, but higher than the job embeddedness level of anesthesiology nurses reported by Yu Yao et al. (4). The work content of physical examination nurses is relatively standardized, primarily involving routine health assessments and consultations, with potentially stable work rhythms and environments. This stability may contribute to a relatively higher level of job embeddedness among these nurses. Additionally, factors such as varying organizational cultures and support systems across different medical institutions, the relative independence of physical examination departments within organizations, or insufficient support and resources may also influence the job embeddedness level of physical examination nurses.

### Heterogeneity exists in the job embeddedness levels among physical examination nurses

The study revealed that the job embeddedness levels of physical examination nurses could be classified into three latent classes: a Low Job Embeddedness Group comprising 40 cases (26.7%), a Moderate Job Embeddedness Group with 47 cases (31.4%), and a High Job Embeddedness Group including 63 cases (41.9%). Heterogeneity was observed among these different categories. Nurses in the low embeddedness group exhibited lower levels of job embeddedness, which may be attributed to factors such as lower income and dissatisfaction with personal career advancement. In contrast, nurses in the moderate and high embeddedness groups demonstrated higher levels of job embeddedness, likely due to stable income, a supportive work environment, and stronger psychological capital. These results are consistent with the findings reported by Ji Ming et al. (13) regarding nurses' work environments and with previously published studies related to psychological capital (14). Consequently, it is suggested that the career advancement pathways for physical examination nurses should be revised, and managers are advised to enhance employee care initiatives.

### Factors influencing the latent profile analysis of job embeddedness levels among physical examination nurses

Univariate analysis revealed that there were statistically significant differences (*p* < 0.05) in the job embeddedness of physical examination nurses across different latent profiles regarding age, childbirth status, annual income, satisfaction with personal promotion, nurse retention intention scores, and PCQ scores. The study indicated that higher average monthly income is associated with a greater degree of job embeddedness among nurses, which aligns with the findings of Wang Yan et al. (15). Additionally, nurse retention intention has been reported as a significant predictor of nurse retention (16). Research by Wang Chen et al. also found that the nursing work environment, satisfaction with personal promotion, psychological capital, and average monthly income are primary factors influencing nurse job embeddedness (17)**.**

Annual income directly reflects both the professional valuation and economic security of nurses. Inadequate income may lead nurses to question organizational fairness and their own sense of professional worth, thereby weakening the financial bond with their work. Only when income reaches a relatively high and competitive level are nurses more likely to develop a strong perception of “economic sacrifice,” which in turn enhances their level of job embeddedness. Therefore, optimizing compensation structures and ensuring income competitiveness are fundamental material safeguards for improving job embeddedness.

High satisfaction with career advancement indicates that nurses perceive a strong alignment between their personal development and that of the organization, making them more willing to invest emotionally and professionally in long-term career growth. By offering transparent, equitable, and predictable career advancement pathways, organizations can significantly enhance nurses' sense of “organizational fit,” thereby deepening their level of job embeddedness.

Retention intention reflects nurses' emotional commitment to the organization as well as the perceived barriers to leaving. Low retention intention often signals a state of “psychological detachment,” which may be linked to work-related stress, burnout, or a lack of belonging. Enhancing retention intention requires improving the work environment, strengthening team support, and fostering emotional connections, thereby helping nurses build robust networks in the “linkage” dimension and reducing their inclination to leave voluntarily. Organizations should recognize the diverse needs of nurses at different life stages and provide targeted support strategies—such as parental leave, flexible scheduling, and career mentorship programs—to indirectly enhance their level of job embeddedness.

A healthy work environment can maximize nurses' enthusiasm and engagement, leading to faster career advancement. Conversely, an unfavorable nursing work environment tends to reduce job satisfaction, increase burnout, and heighten turnover risk (18, 19), thereby hindering the formation of close organizational ties. Nurses with high retention intention exhibit greater motivation and engagement at work, allowing positive retention factors to function more effectively and resulting in higher levels of job embeddedness.

This study further found that higher scores on psychological capital traits are associated with deeper job embeddedness among nurses. In nursing practice, a strong sense of self-efficacy enables nurses to believe in their ability to effectively perform tasks, including patient care and emergency response. Consequently, nurses with high self-efficacy often demonstrate greater job embeddedness, set clear career goals, and persistently strive toward them. Driven by such positive emotions, nurses become more immersed in their work and perceive a stronger connection between their professional duties and personal growth, thereby elevating their level of job embeddedness.

However, this study also has several limitations. The cross-sectional design adopted here only reflects variable relationships at a specific time point, making it impossible to infer causality or observe dynamic trends. Second, although the sample size met statistical requirements, all participants were recruited from a single institution. Factors such as regional culture and management systems may limit the generalizability of the findings to other regions or hospital levels. Future research should expand the scope and diversity of the sample to improve external validity.

Data were primarily collected through self-reported questionnaires, which may be subject to common method bias and social desirability effects. Future studies could incorporate qualitative interviews or objective behavioral indicators for multi-source validation. The research focused on individual and organizational factors, without fully examining the potential moderating effects of external environmental variables such as macro-level policies or healthcare system reforms. Subsequent studies could include broader contextual variables to develop a more integrated theoretical model.

Despite these limitations, this study, through latent profile analysis, revealed heterogeneous patterns of job embeddedness among physical examination nurses, providing empirical evidence for targeted management strategies. Future research may further explore underlying mechanisms using mixed-methods approaches.

This study systematically examined key variables influencing the level of job embeddedness among nurses in health examination departments and identified several statistically significant predictors. Specifically, satisfaction with career advancement, annual income, turnover intention, and psychological capital were confirmed as core factors contributing to enhanced job embeddedness.

From a theoretical perspective, the findings extend the application of job embeddedness theory within the nursing profession, providing empirical evidence on how structural factors (e.g., promotion mechanisms, income levels) and psychological factors (e.g., turnover intention, psychological capital) jointly influence nurses' career stability.On a practical level, this study offers evidence-based insights to support healthcare institutions in developing more targeted human resource policies. Transparent and equitable promotion mechanisms, along with industry-competitive compensation structures, should be established to directly address nurses' core needs regarding satisfaction with advancement and reasonable income, thereby strengthening their organizational commitment. Healthcare institutions may implement resilience training, stress management programs, and similar initiatives to systematically enhance nurses' psychological capital, enabling them to better cope with compassion fatigue and work-related stress and, in turn, reinforcing their intrinsic motivation to remain in their roles. Managers should foster a fair and respectful work environment, recognize nurses' professional value, and provide clear career development pathways to significantly enhance their professional identity and retention willingness. These measures can translate the concept of job embeddedness from a theoretical framework into actionable organizational practices.

In summary, improving job embeddedness among health examination nurses requires a coordinated dual-path approach: institutional safeguards (promotion and income) and psychological empowerment (capital and intention). It is recommended that healthcare institutions integrate these key variables into the core monitoring and intervention indicators of their nursing talent retention strategies. Through data-driven, refined human resource management, sustained enhancement of nurse engagement and retention rates can ultimately be achieved.

## Data Availability

The original contributions presented in the study are included in the article/Supplementary Material, further inquiries can be directed to the corresponding author.
